# Significant change of cytochrome P450s activities in patients with hepatocellular carcinoma

**DOI:** 10.18632/oncotarget.9437

**Published:** 2016-05-18

**Authors:** Jun Zhou, Qiang Wen, Sai-Fei Li, Yun-Fei Zhang, Na Gao, Xin Tian, Yan Fang, Jie Gao, Ming-Zhu Cui, Xiao-Pei He, Lin-Jing Jia, Han Jin, Hai-Ling Qiao

**Affiliations:** ^1^ Institute of Clinical Pharmacology, Zhengzhou University, Zhengzhou, People's Republic of China; ^2^ Henan Provincal People's Hospital, Zhengzhou, People's Republic of China

**Keywords:** hepatocellular carcinoma, cytochrome P450, human liver microsomes, drug metabolism, gene polymorphism

## Abstract

The lack of information concerning individual variation in drug-metabolizing enzymes is one of the most important obstacles for designing personalized medicine approaches for hepatocellular carcinoma (HCC) patients. To assess cytochrome P450 (CYP) in the metabolism of endogenous and exogenous molecules in an HCC setting, the activity changes of 10 major CYPs in microsomes from 105 normal and 102 HCC liver tissue samples were investigated. We found that CYP activity values expressed as intrinsic clearance (CL_int_) differed between HCC patients and control subjects. HCC patient samples showed increased CL_int_ for CYP2C9, CYP2D6, and CYP2E1 compared to controls. Meanwhile, CYP1A2, CYP2C8, and CYP2C19 CL_int_ values decreased and CYP2A6, CYP2B6, and CYP3A4/5 activity was unchanged relative to controls. For patients with HCC accompanied by fibrosis or cirrhosis, the same activity changes were seen for the CYP isoforms, except for CYP2D6 which had higher values in HCC patients with cirrhosis. Moreover, CYP2D6*10 (100C>T), CYP2C9*3 (42614 A>C), and CYP3A5*3 (6986A>G) polymorphisms had definite effects on enzyme activities. In the HCC group, the CL_int_ of CYP2D6*10 mutant homozygote was decreased by 95% compared to wild-type samples, and the frequency of this homozygote was 2.8-fold lower than the controls.

In conclusion, the activities of CYP isoforms were differentially affected in HCC patients. Genetic polymorphisms of some CYP enzymes, especially CYP2D6*10, could affect enzyme activity. CYP2D6*10 allelic frequency was significantly different between HCC patients and control subjects. These findings may be useful for personalizing the clinical treatment of HCC patients as well as predicting the risk of hepatocarcinogenesis.

## INTRODUCTION

Hepatocellular carcinoma (HCC) is the most common form of liver cancer and a leading cause of cancer death worldwide [1, 2]. HCC is often accompanied by severe liver dysfunction that is closely related to disease progression, which can influence HCC treatment decisions. HCC pathogenesis involves multiple factors [3] and is often promoted by chronic infection with the hepatitis virus, especially hepatitis B and C virus (HBV and HCV) [2]. A decrease in carcinogen metabolism and an increase in procarcinogen activation have also been documented as HCC risk factors as well as changes in the metabolism of environmental toxins that arise from alterations in CYP activity [4–7].

Cytochrome P450 (CYP) is a large group of enzymes that localize to mitochondrial membranes or the endoplasmic reticulum and play crucial roles in the metabolism of endogenous and exogenous molecules, including most drugs [8]. CYP1, 2, and 3 families are responsible for the metabolism of the majority of drugs and other xenobiotics [9]. The most significant drug metabolizing CYPs in humans include CYP1A2, CYP2A6, CYP2B6, CYP2C8, CYP2C9, CYP2C19, CYP2D6, CYP2E1, and CYP3A4/5, which are responsible for the metabolism of approximately 90% of drugs used clinically and/or metabolic activation of procarcinogens [9–11]. CYP enzymes are key enzymes involved in cancer development [12]. They mediate the metabolic activation of numerous procarcinogens [12]. Therefore, assessment of changes in CYP enzyme activity would be useful not only for designing personalized HCC treatments, but also for identifying potential factors that contribute to HCC susceptibility. Due to numerous physiological disorders that can accompany HCC, treatment of this cancer type and its complications often requires adjustment of drug dosages. However, using changes in CYP enzyme activity in HCC patients as the basis of dosage adjustment has not been described to date.

Yan et al. investigated the activity of seven CYPs in tumors obtained from 26 patients with HCC [13]. The results showed that activity of these seven CYP isoforms was decreased in tumor human liver microsomes (HLMs) relative to those in pericarcinomatous HLMs and control HLMs. The pericarcinomatous tissues were confirmed to be cirrhosis, however the disease history of the donors of the control pooled HLMs was not clear. Another similar study characterized CYP3A4 activity in tumor tissue from 18 HCC patients [14]. The liver microsomes from adjacent non-cancerous tissue and pooled male liver microsomes were used as controls. In these two reports the individual variation of CYP activity in normal liver tissue was not described.

Early *in vitro* studies have indicated that CYPs activity is selectively altered in liver undergoing cirrhosis [15–18]. In addition, some *in vivo* studies have reported that CYP isoform activity is differentially affected by the presence of liver disease [19–21]. However, these studies focused on simple cirrhosis without accompanying HCC and therefore the results may be inappropriate for use in designing personalized approaches for treating HCC patients.

Since all CYPs that metabolize drugs are polymorphic, numerous studies have focused on the relationship between the distribution of mutant CYP alleles and risk of developing different types of cancer. DNA mutations are known to be important carcinogenic factors in many tumor-related diseases [22]. However, a consistent consensus does not as yet exist regarding causation. Some *in vivo* studies suggest that CYP polymorphisms maybe related to HCC, while others found that CYP mutations had no effect [12, 23–27]. Due to these contradictory conclusions, exploring the relationship between CYP polymorphisms and drug-metabolizing changes in liver cancer patients is important for providing personalized treatment options.

The present study systematically assessed changes in ten major CYP enzymes in HLMs from HCC patients compared to normal liver tissue. The effect of HCC together with liver disease (i.e., cirrhosis and fibrosis) and CYP single nucleotide polymorphisms (SNPs) was also evaluated. Twenty-four CYP SNPs with frequencies of more than 1% in Chinese people were investigated. These mutant sites could represent the main SNPs that affect metabolic enzyme activities. We believe that our data show inter-individual differences in CYP metabolism, which can be used for a personalized treatment approach of HCC.

## RESULTS

### Clinical characteristics of patients

Clinical, laboratory, and demographic data were collected from 102 HCC patients and 105 control subjects (Table 1). In both groups, over half the subjects were between 45 and 59 years old. There were 54 patients with liver fibrosis (including S1, S2, and S3) and 48 patients with liver cirrhosis (S4) among the HCC patients. Compared with controls, there were more males, smokers, and drinkers in the HCC group (*P* < 0.01). The age of the HCC subjects was slightly older than the controls (*P* < 0.05). All subjects only received regular anesthetics and had no history of exposure to known CYP-inducing or -inhibiting agents.

**Table 1:** The basic clinical characteristics in HCC patients (n=102) and control subjects (n=105)

Variables	Control	HCC	*P*-value
Gender			<0.01
Male (n, %)	37 (35.2%)	86 (84.3%)	
Female (n, %)	68 (64.8%)	16 (15.7%)	
Smoking			<0.01
Yes (n, %)	12 (11.9%)	45 (44.1%)	
No (n, %)	89 (88.1%)	57 (55.9%)	
Drinking (n, %)			<0.01
Yes	12 (11.9%)	37 (36.3%)	
No	89 (88.1%)	65 (63.7%)	
Age			<0.05
Average ± S.D. (years)	48 ± 10	53 ± 11	
< 44 year (n, %)	35 (33.3%)	21 (20.6%)	
45-59 year (n, %)	56 (53.3%)	51 (50.0%)	
60-74 year (n, %)	13 (12.4%)	29 (28.4%)	
> 75 year (n, %)	1 (1.0%)	1 (1.0%)	
HbsAg anti-HCV Pathologyclassification	Negative (n=105) Negative (n=105) Normal (n=105)	Positive (n=96) Positive (n=6) S1, S2, S3, S4.	
Diagnosis	liver hemangioma (n=84) metastatic carcinoma (n=8) cholelithiasis (n=9) gallbladder cancer (n=4)	HCC (n=102)	

S1: portal area fibrosis expand, and confined in the hepatic sinus and lobule (n=8); S2: fibrosis around portal area, fibrous septums form, lobule structure keep (n=19); S3: fibrous septums and lobule structure disorder, without cirrhosis (n=27); S4: early stage cirrhosis (n=48). HCC indicates hepatocellular carcinoma. n=number. *P* value for comparison between the controls and HCC sets.

### Enzyme activity in HLMs

**Figure 1:**
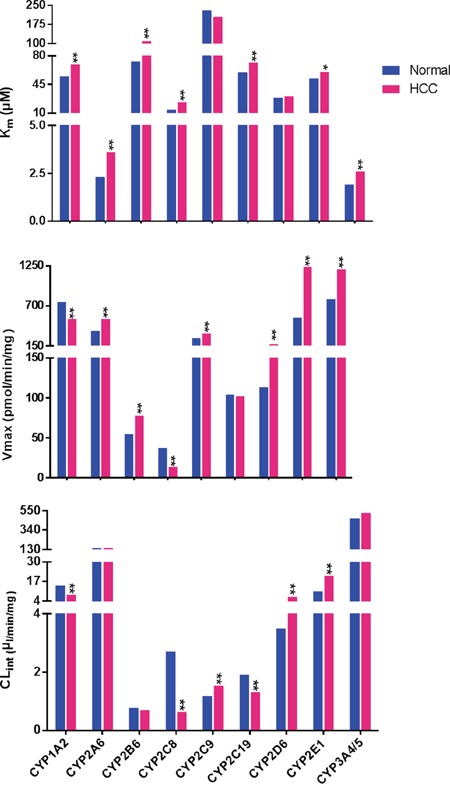
Inter-individual enzyme activities in 102 hepatocellular carcinoma (HCC) HLMs and 105 control HLMs Enzyme activities are expressed as the median values. Blue bars represent enzyme activity in control HLMs. Red bars represent those from HCC group. “*”, “**” denote significant differences from controls (*P* < 0.05 and *P* < 0.01, respectively) by the Mann-Whitney U test.

The median individual metabolic parameters (K_m_, V_max_, CL_int_) of liver microsomes from both HCC groups and controls were determined (Figure 1). Compared with controls, the K_m_ values for CYP1A2, CYP2A6, CYP2B6, CYP2C8, CYP2C19, CYP2E1, and CYP3A4/5 were higher in HCC patients. Meanwhile, CYP2C9 and CYP2D6 values did not differ between HCC patients and control subjects. CYP2A6, CYP2B6, CYP2C9, CYP2D6, CYP2E1, and CYP3A4/5 had higher V_max_ values in HCC patients, while the V_max_ values of CYP1A2 and CYP2C8 were significantly reduced relative to control samples. The V_max_ of CYP2C19 showed no difference between HCC patients and control subjects. The most notable difference between the two groups was the V_max_ of CYP2E1, which was increased by 2.3-fold in HCC patients relative to controls (1238.0 vs. 539.3 pmol/min/mg, respectively; *P* < 0.01). The CL_int_ values for CYP2C9, CYP2D6, and CYP2E1 were increased in HCC patients, while CYP1A2, CYP2C8, and CYP2C19 values were lower in HCC patients than in controls. The CL_int_ values for CYP2A6, CYP2B6, and CYP3A4/5 were unchanged between the two groups. The most prominent change was the CL_int_ of CYP2C8, which in HCC patients was 77% that of the value for the controls (0.63 vs. 2.7μl/min/mg, respectively; *P* < 0.01).

Together these results showed that the enzyme activities of the 10 CYP isoforms selectively varied in HCC patient samples.

### Effect of fibrosis and cirrhosis on enzyme activity

In order to further evaluate the effect of disease progression on CYP activities, HCC patients were categorized into two subgroups that included patients with fibrosis or cirrhosis.

**Figure 2:**
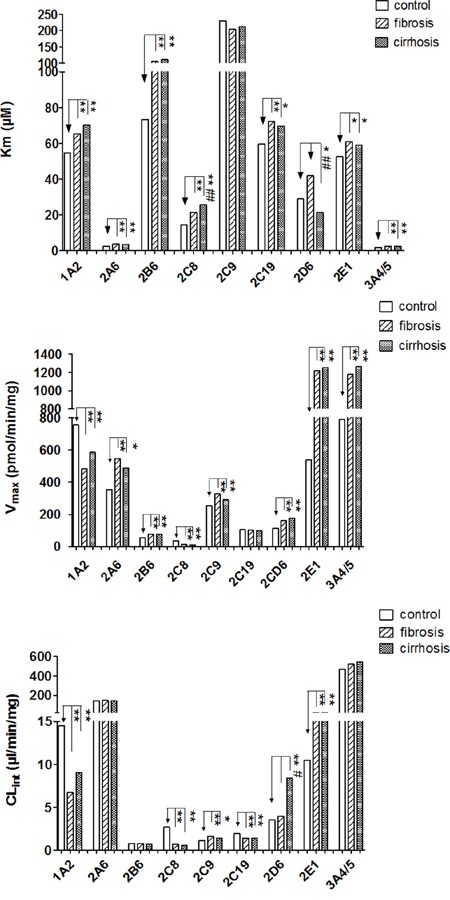
The km, Vmax and CLint values of 10 CYPs in the fibrosis group, cirrhosis group, and control subjects Data are shown as bar graphs representing medians. Blank bars represent enzyme activity in controls. Stripe bars represent enzyme activity from the fibrosis group. Dot bars represent enzyme activity in the cirrhosis group. “*” and “**” denote differences that are significantly different from controls (*P* < 0.05 and *P* < 0.01, respectively), “^#^”, “^##^” denote significant differences from the fibrosis group (*P* < 0.05 and *P* < 0.01, respectively) by the Mann-Whitney U test.

As shown in Figure 2, the K_m_ values of most CYP isoforms did not differ between the fibrosis and cirrhosis subgroups with the exception of CYP2D6 and CYP2C8. The K_m_ of CYP2C8 was higher (25.7 vs. 21.4 μM; *P* < 0.05), but the value of CYP2D6 was lower (21.4 vs. 41.9 μM; *P* < 0.01) in the cirrhosis group relative to the fibrosis group, respectively. In terms of V_max_, the CYP isoform activities were not different between the fibrosis and cirrhosis groups. The presence of fibrosis or cirrhosis significantly affected the CYP2D6 CL_int_ value only, which was increased by 53.6% in patients with cirrhosis relative to those with fibrosis (8.4 vs. 3.9 μM, respectively; *P* < 0.05).

Compared with controls, the K_m_ values of CYP1A2, CYP2A6, CYP2B6, CYP2C8, CYP2C19, CYP2E1, and CYP3A4/5 were higher in both in fibrosis and cirrhosis groups. CYP2C9 K_m_ was not changed relative to controls. In terms of V_max_, CYP2A6, CYP2B6, CYP2C9, CYP2D6, CYP2E1, and CYP3A4/5 were increased, while the V_max_ values of CYP1A2 and CYP2C8 were decreased in both the cirrhosis and fibrosis groups compared to control subjects. CYP2C19 V_max_ was not different compared to controls. The CL_int_ values of CYP1A2, CYP2C8, and CYP2C19 were decreased, while CYP2C9 and CYP2E1 values were increased. In addition, the CL_int_ values of CYP2A6, CYP2B6, and CYP3A4/5 were unchanged relative to controls. Interestingly, the change in CYP2D6 activity between the cirrhosis and fibrosis groups was not consistent. The presence of cirrhosis significantly affected CYP2D6 activity by causing a decrease in the K_m_ value (21.4 vs. 28.9μM, respectively; *P* < 0.05) and an increase in the CL_int_ value (8.4 vs. 3.5 μl/min/mg, respectively; *P* < 0.01) relative to controls. However, the K_m_ and CL_int_ values of CYP2D6 in the fibrosis group did not significantly differ from the control subjects (Figure 2).

Taken together, these results indicate that the effect of disease progression on CYP enzyme activities were not significantly different in HCC patients with fibrosis and cirrhosis, with the exception of CYP2D6.

### Allele and genotype frequency distribution

The allelic frequency of CYP2D6*10 (100C>T) significantly differed between the control and HCC groups (Table 2). Moreover, the CYP2D6*10 mutant homozygote *T/T* frequency was lower (16.7% vs. 46.7%; *P* < 0.01) and the heterozygote *C/T* frequency was significantly higher (46.9% vs. 21.9%; *P* < 0.01) in HCC groups relative to control subjects, respectively. Among control group subjects, the highest genotype frequency was CYP2D6*10 T/T, which had the lowest frequency in the HCC group.

**Table 2:** The genotype frequency of CYP2D6*10 (100C>T) in controls, HCC, fibrosis and cirrhosis groups

Genotype	Control		HCC		Fibrosis		Cirrhosis	
	n	Freq (%)	n	Freq (%)	n	Freq (%)	n	Freq (%)
C/C	33	31.4	35	36.5	18	35.3	17	37.8
C/T	23	21.9	45	46.9**	21	41.2**	24	53.3**
T/T	49	46.7	16	16.7**	12	23.5**	4	8.9**##
Total	105		96		51		45	

** *P* < 0.01 vs. controls.

## *P* < 0.01 vs. fibrosis group.

HCC=hepatocellular carcinoma. n=number. Freq = genotype frequency.

The allelic frequency of the CYP2D6*10 mutation was significantly reduced in HCC patients with fibrosis or cirrhosis relative to controls. The mutant homozygote *T/T* frequency was also lower in both in fibrosis and cirrhosis groups than control subjects. In contrast, the frequency of *C/T* was increased in both the fibrosis and cirrhosis groups compared to control subjects.

In addition, the frequency of mutant homozygote *T/T* was significantly reduced in the cirrhosis group relative to the fibrosis group (8.9% vs. 23.5%, respectively; *P* < 0.01).

The allelic frequencies of CYP2C9*3 and CYP3A5*3 were not different in HCC patients (Supplementary Table S3).

### Effect of CYP genetic polymorphisms on enzyme activity

**Figure 3:**
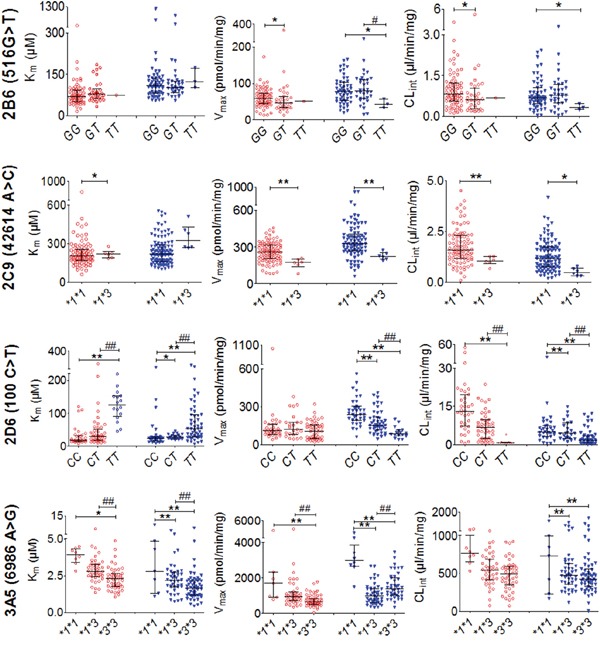
Inter-individual and inter-genotype enzyme activities in 102 hepatocellular carcinoma (HCC) samples and 105 control samples Red open circles (○) represent enzyme activity in control HLMs; Blue triangles (▼) represent those from the HCC group. Horizontal lines indicate median with inter-quartile range. “*”, “**” denote differences that are significantly different from wild-type (*P* < 0.05 and *P* < 0.01, respectively), “^#^”, “^##^” indicate significant differences from the mutant heterozygote (*P* < 0.05 and *P* < 0.01, respectively) by the Mann-Whitney U test.

Twenty-four DNA polymorphisms were analyzed in HCC patients and controls. The polymorphisms that influenced enzyme activity are shown in Figure 3. In the HCC group, the K_m_ of the CYP2D6*10 mutant homozygote *T/T* (125.1 μM) was significantly higher than that for the heterozygote *C/T* (30.4 μM) and wild-type *C/C* (17.4 μM). The pattern for V_max_ values differed from that for K_m_ values, with *C/C* (241.5 pmol/min/mg) being the highest and *T/T* (89.4 pmol/min/mg) being the lowest, while *C/T* (155.6 pmol/min/mg) had intermediate activity. The CL_int_ of *T/T* (0.70 μl/min/mg) was remarkably lower than that for *C/T* (6.9 μl/min/mg) or *C/C* (13.2 μl/min/mg). The most significant change in CL_int_ was for *T/T*, which was decreased by 95% compared with *C/C*.

The K_m_ of CYP3A5*3 mutant homozygote *3/*3 (2.3 μM) was significantly lower than in the wild-type *1/*1 (3.9 μM) and mutant heterozygote *1/*3 (2.8μM). The V_max_ showed the same differences as K_m_ (*3/*3<*1/*3<*1/*1, 994.8 vs. 1402.5 vs. 2975.5 pmol/min/mg, respectively). Similarly, the CL_int_ of *3/*3 (490.8 μl/min/mg) and *1/*3 (542.0 μl/min/mg) both were significantly lower than *1/*1 (762.9 μl/min/mg).

The CYP2C9*3 mutant homozygote *3/*3 was not detected in HCC patients. The V_max_ of the mutant heterozygote *1/*3 was significantly lower than that for wild-type *1/*1 (223.2 vs. 326.5 pmol/min/mg, respectively; *P* < 0.01). Meanwhile, the CL_int_ of *1/*3 (1.1 pmol/min/mg) was significantly lower than for*1/*1 (1.6 pmol/min/mg, *P* < 0.05).

In addition, the CL_int_ and V_max_ values of *CYP2B6* (516G>T) mutant homozygote *T/T* were lower than those of wild-type *G/G*. No significant influence on enzyme activity was identified for the other CYP polymorphisms assessed.

In the control group, similar result was observed. CYP2D6*10, CYP3A5*3 and CYP2C9*3 mutations had clear effects on enzyme activities.

### Effect of demographic factors and clinical data on CYP activity

**Figure 4:**
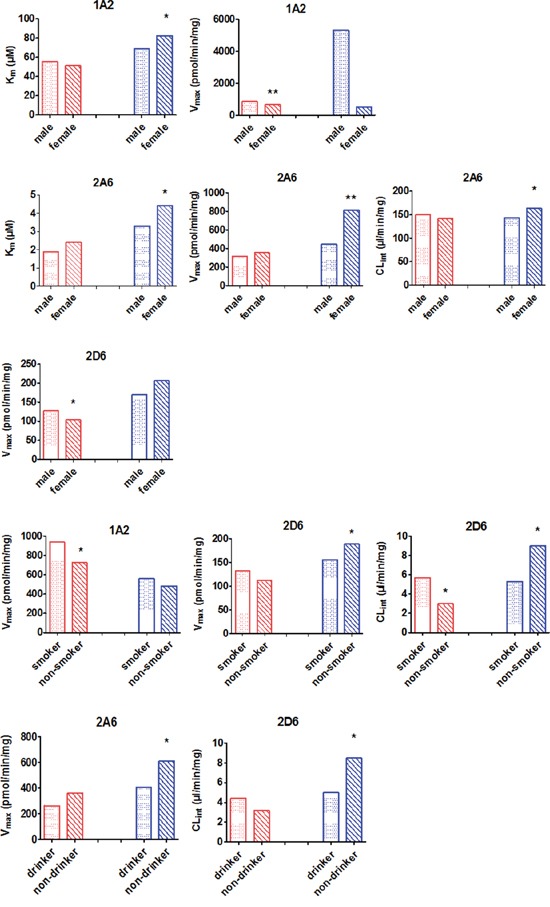
Effect of demographic factors on CYP activity Data are shown as columns representing median individual metabolic parameters. Red bars represent the enzyme activity in controls. Blue bars represent the enzyme activity from the HCC group. “*” and “**” indicate significant differences from male/smoker/drinker by the Mann-Whitney U test.

The influence of sex, tobacco smoking, and alcohol consumption were also analyzed and the result was shown in Figure 4.

Using CL_int_ values as a measure of CYP activity, we found that females with HCC had higher CYP2A6 CL_int_ values than male HCC patients (164.1 vs. 142.1 μl/min/mg, respectively; *P* < 0.05). The CL_int_ for CYP2D6 was higher for HCC patients who were non-smokers than for HCC patients who smoked (9.0 vs. 5.3 μl/min/mg, respectively; *P* < 0.05). Similarly, the CL_int_ of CYP2D6 for HCC patients who did not drink was higher than for HCC patients who did drink (8.5 vs. 5.0 μl/min/mg, respectively; *P* < 0.05).

In control subjects, there were no statistically significant differences (*P* > 0.05) in the CL_int_ of 10 CYPs as a function of gender, smoking status, or drinking habit with the exception of CYP2D6. History of smoking was associated with significant differences in the CL_int_ value of CYP2D6 compared to controls (3.0 vs. 5.7μl/min/mg, respectively; *P* < 0.05).

## DISCUSSION

In this study we found varying activities of 10 CYP isoforms in HLMs isolated from HCC patients compared to normal control HLMs, which were obtained from patients with hemangioma or other liver diseases and confirmed by histopathological examination and a liver function test. In HCC patients, CL_int_ values increased for CYP2C9, CYP2D6, and CYP2E1, while the values for CYP1A2, CYP2C8, and CYP2C19 decreased. In addition, CYP2A6, CYP2B6, and CYP3A4/5 activities were unchanged between HCC patients and control subjects. Diseases that often accompany HCC, such as fibrosis and cirrhosis, did not affect CYP activity changes, except for CYP2D6 which had higher CL_int_ values in the presence of cirrhosis. Moreover, gene polymorphisms and genotype frequency of some CYP isoforms, especially CYP2D6*10, clearly affected CYP activity in HCC patients.

Given the important role of CYP isoforms in drug metabolism, these HCC-related changes in CYP activity could influence the pharmacokinetics of drugs used to treat this cancer. Several previous studies reported that the activities of several CYPs, including CYP1A2, CYP2A6, CYP2D6, CYP2C19, CYP2E1, and CYP3A were decreased in cirrhosis patients relative to healthy subjects [15, 16, 19-21, 31-35]. However, in the current study we found that the CYP2D6 CL_int_ value from the cirrhosis patient subgroup was significantly higher than that for HCC patients without cirrhosis. This inconsistency could in part be due to the fact that these earlier studies focused on patients with simple cirrhosis who did not have accompanying HCC. Meanwhile, another study found that CYP2A6 activity decreased in patients with either moderate or severe alcoholic liver disease, but not in those with only mild forms of the disease [31]. In contrast, our study showed that CYP2A6 activity was not changed in the fibrosis or cirrhosis subgroups of HCC patients, which again demonstrates the differences that may exist between simple cirrhosis and cirrhosis that accompanies HCC. The different characteristics identified in our study suggest that a special reference standard would be essential for personalizing treatments for HCC patients with cirrhosis.

Some exogenous substances can be metabolized by CYP into carcinogenic molecules. For example, nitrosamine is a common procarcinogen that is metabolized by CYP2E1 into acetaldehyde, which can induce multiple cancer types. Our data showed that CYP2E1 activity was higher in HCC patients. However, a previous study found that CYP2E1 activity was lower in patients with moderate or severe cirrhosis [17]. The differences may again be due to the different inclusion criteria. Our study considered patients with cirrhosis or fibrosis that accompanied HCC, while the other study examined only those patients with simple cirrhosis [17]. Therefore, additional studies are needed to confirm whether changes in CYP2E1 activity are critical factors in the progression of cirrhosis to HCC. However, the increased levels of pre-carcinogen activation might explain in part why CYP2E1 activities were higher in HCC patients than in simple cirrhosis patients. The results also indicate that CYP activity might be a risk factor for HCC and a candidate marker for metabolic susceptibility to HCC.

Although numerous studies have reported that DNA sequence mutations are associated with HCC susceptibility [23-27, 36, 37], in this study we found that only the allelic frequency distributions of CYP2D6*10 significantly differed between the control and HCC groups. Moreover, in both the control and the HCC group, the CYP2D6*10 T/T type carriers had lower CL_int_ than *C/C* or *C/T* carriers. Relative to control subjects, the *T/T* frequency was lower in HCC patients, especially in the cirrhosis subgroup. This result is consistent with reports by Silvestri et al. and Agúndez et al. [24, 25]. The higher CYP2D6 activity in HCC patients compared to control subjects may be attributed to them carrying more functional alleles. In addition, subjects who carried the CYP2D6*10 mutant homozygote appeared to have a lower risk of developing HCC.

In order to further verify the influence of these factors on CYP activity in HCC patients, we investigated the effect of demographics data on CYP activities. CYP activity values are expressed as intrinsic clearance (CL_int_), we did not observe an obvious effect of gender, smoking, or drinking on most CYP activities, with the exception of CYP2D6 and CYP2A6. The CL_int_ for CYP2D6 was lower in HCC patients who were smokers and drinkers compared to those who were non-smokers and non-drinkers. In this study there were more smokers and drinkers in the HCC group than in the control group and CYP2D6 CL_int_ was higher in HCC patients than in control subjects. Therefore, smoking may not be related to the activity changes observed in HCC patients, or at least not the main influencing factor. In addition, males had a lower CL_int_ value of CYP2A6 than females in the HCC group. However, there were more males in the HCC patient group compared to the control group and the CYP2A6 CL_int_ value did not differ between these two groups. Therefore, gender may not have a substantial impact on CYP activity. Previous studies demonstrated that smoking is clinically induction of liver microsomal CYP1A2 [35]. Alcohol consumption is known to cause induction of liver microsomal CYP2E1 [38]. In the present study, it was not observed the effect of tobacco smoking and alcohol consumption on CYP1A2 and CYP2E1 activities. This disparity may due to other factor, including ethnic, regional, disease, and sample number.

In conclusion, the 10 tested CYP isoforms exhibited different changes in activity in HCC patients as evidenced by increased, decreased, and constant CL_int_ values relative to control subjects. In addition, accompanying symptoms of fibrosis and cirrhosis affected CYP activities differently in HCC patients. These results indicate that some therapeutic regimens, including drugs metabolized by CYP isoforms, may require adjustments in HCC patients. Our observations in this study may be helpful for designing personalized treatments for HCC patients. In addition, CYP2D6*10 had unique gene polymorphisms and genotype frequency, and the mutation clearly affected enzyme activity in HCC patients. Moreover, the activity of CYP2E1, which is the major enzyme that metabolizes ethanol and nitrosamine, was significantly increased in HCC patients compared to that in the control subjects. These data could also be helpful for understanding disease mechanisms in HCC patients. Our findings suggest that the polymorphism of CYP2D6*10 may be a good predictor of hepatocarcinogenesis.

## MATERIALS AND METHODS

### Ethical Statement

Approvals for tissue collection and *in vitro* xenobiotic metabolism studies were obtained from the Medical Ethics Committee of Zhengzhou University. Informed written consent was obtained from the patients for the collection of liver specimens.

### Human Liver Samples

All non-tumor liver tissues were obtained from members of the Chinese Han population who underwent surgical resection in the Henan Provincal People's Hospital, the First Affiliated Hospital of Zhengzhou University, and Henan Provincal Tumor Hospital between March 2012 and July 2014. All patients received a liver function test, histopathological analysis, and imaging (ultrasonography or computer tomography). All liver samples from patients with tumors were 2 cm distant from the tumor tissue and were stored in liquid nitrogen within 30 min of resection until use.

HBV- or HCV-infected liver tissues (102 samples) were all from patients with HCC. Liver samples were categorized as having fibrosis or cirrhosis by histopathological examination. Liver tissue samples (105 samples) were collected from subjects with liver hemangioma, metastatic carcinoma, cholelithiasis, or gallbladder cancer. These subjects had normal liver function and negative serum HBV and HCV markers, and the liver samples were confirmed to be normal by histopathological examination. These samples were used as normal liver controls.

Detailed information for each patient was well-documented and included gender, age, body height, body weight, smoking habits, alcohol consumption, clinical diagnosis, regular drug intake before surgery, previous history, allergic history, pathological diagnosis, imaging examination, and laboratory test data (including, but not limited to, results from routine blood analysis, liver function tests, and renal function tests). We defined ‘drinkers’ as those who have drunk 2-3 times or more drinking per week, ‘non-drinkers’ as individuals who had never drunk, or those who had drunk less than 2 times per week. We defined ‘smokers’ as those who smoke 11 cigarettes or more smoking per day, ‘non-smokers’ as individuals who had never smoked or smoke less than 11 cigarettes per day. The standard referred the previous method [28].

### Preparation of liver microsomes

Human liver microsomes (HLMs) were prepared according to the hypothermal differential centrifugation method as previously described [29]. Microsomal protein concentration was determined according to the Bradford method [30].

### Enzyme activities in HLMs

All probe drugs and part metabolites used in the current study were purchased from the National Institute for the Food and Drug Control (China), including phenacetin, acetaminophen, coumarin, bupropion, paclitaxel, tolbutamide, omeprazole, dextromethorphan, chlorzoxazone, and midazolam. Other metabolites (7′-hydroxycoumarin, hydroxybupropion, 6-hydroxypaclitaxel, 4′-hydroxytolbutamide, 5′-hydroxyomeprazole, O-demethylation dextrorphan, 6-hydroxychlorzoxazone, and 1′-hydroxymidazolam) were purchased from Sigma-Aldrich (St. Louis, MO, USA). Reduced nicotinamide adenine dinucleotide phosphate (NADPH) was obtained from Roche Co., Ltd. (Switzerland). All organic solvents were of high performance liquid chromatography (HPLC) purity and obtained from Siyou Chemical Reagent Co. (Tianjin, China).

The activity of the 10 most important CYP isoforms, including CYP1A2, CYP2A6, CYP2B6, CYP2C8, CYP2C9, CYP2C19, CYP2D6, CYP2E1, and 3A4/5, were measured using HLMs and probe substrates. Phenacetin O-deethylation, coumarin 7-hydroxylation, bupropion 4-hydroxylation, paclitaxel 6-hydroxylation, tolbutamide 4-hydroxylation, omeprazole 5-hydroxylation, dextromethorphan O-demethylation, chlorzoxazone 6-hydroxylation, and midazolam 1-hydroxylation were used as activity indicators of CYP1A2, CYP2A6, CYP2B6, CYP2C8, CYP2C9, CYP2C19, CYP2D6, CYP2E1, and CYP3A/5, respectively.

For biotransformation, seven or eight substrate concentrations were examined. Incubation mixtures contained HLMs, 100 mM phosphate buffer (pH 7.4), and different concentrations of substrate with 1 mM NADPH. The mixture was pre-incubated for 5 min at 37°C. Reactions were terminated by adding 20μl ice-cold acetonitrile, 1 ml ethylacetate, or 10 μl perchloric acid. The detailed description of incubation conditions for the 10 CYP activity assays is provided in Supplementary Table S1.

The formation of all substrate metabolites was determined by HPLC. Several substrate metabolites, including acetaminophen, hydroxybupropion, 6-hydroxypaclitaxel, 4′-hydroxytolbutamide, 5′-hydroxyomeprazole, 6-hydroxychlorzoxazone, and 1′-hydroxymidazolam, were separation by HPLC-UV. The 7′-hydroxycoumarin and O-demethylation dextrorphan were separation by HPLC-FLD. A detailed description of the separation methods used for the 10 CYP activity assays is provided in Supplementary Table S2.

### Genotyping

Genomic DNA was isolated from human liver tissues using a genomic DNA purification kit (QIAGEN Translational Medicine Co., Ltd, China). Polymorphisms in 10 CYP isoforms with frequencies of more than 1% in the Chinese sample set were investigated. A total of 23 allelic mutations were determined by mass spectrometry performed by the LIUHE HUADA Genomics Technology Co., Ltd. (Beijing, China). And the allele CYP2E1*1C/*1D were genotyped using one-step PCR.

### Statistical analysis

Since most data sets were not normally distributed, nonparametric methods were generally used for statistical analyses. The Michaelis-Menten constant (K_m_) and maximum reaction velocity (V_max_) values were determined using GraphPad Prism 5.0. *In vitro* intrinsic clearance (CL_int_) was calculated from the ratio of V_max_ to K_m_. SPSS statistics17.0 software was used for statistical analyses. The Mann-Whitney U test was used for pairwise comparisons. *P* values<0.05 were considered statistically significant (two-tailed). All graphs were generated using the Adobe Photoshop CC 2014, PowerPoint 2016 and GraphPad Prism version 5.0 software package.

## SUPPLEMENTARY MATERIALS TABLES


