# Supplementary material for: Significant change of cytochrome P450s activities in patients with hepatocellular carcinoma
Source: Oncotarget. 2016 May 18;7(31):50612–23. doi: 10.18632/oncotarget.9437 (PMC5226607; doi:10.18632/oncotarget.9437)
Supplement: Supplementary file 1 [file oncotarget-07-50612-s001.pdf]

Significant change of cytochrome P450s activities in patients with hepatocellular carcinoma

SUPPLEMENTARY TABLES

Supplementary Table S1: Incubation conditions for 10 CYPs activity assays

CYP isoform	Probe substrate	Substrate range (µM)	HLM protein concentration (mg/ml)	Incubation time (min)	Extraction method	Injection volume (µl)
CYP1A2	Phenacetin	6.25-800	0.3	30	Add 20 µl ice-cold acetonitrile precipitation	80
CYP2A6	Coumarin	0.156-20	0.3	30	Add 10 µl perchloric acid precipitation	80
CYP2B6	Bupropion	7.8-500	0.5	60	Add 10 µl perchloric acid precipitation	80
CYP2C8	Paclitaxel	2.5-80	0.5	120	Add 1 ml ethylacetate extraction	70
CYP2C9	Tolbutamide	31.25-2000	0.5	60	Add 10 µl perchloric acid precipitation	80
CYP2C19	Omeprazole	3.9-500	0.5	90	Add 20 µl ice-cold acetonitrile precipitation	70
CYP2D6	Dextromethorphan	0.625-960	0.2	20	Add 10 µl perchloric acid precipitation	50
CYP2E1	Chlorzoxazone	7.8-1000	0.3	30	Add 1 ml ethylacetate extraction	60
CYP3A4/5	Midazolam	3.9-200	0.2	5	Add 20 µl ice-cold acetonitrile precipitation	50

Supplementary Table S2: The separation methods for 10 CYPs activity assays

CYP isofroms	Analyte	Detection method	Determine wavelength (nm)	Mobile phase (A/B)
CYP1A2	Acetaminophen	HPLC-UV	257	methanol/water = 55/45
CYP2A6	7-hydroxycoumarin	HPLC-FLD	Ex=338, Em=458	methanol/perchloric acid = 55/45
CYP2B6	hydroxybupropion	HPLC-UV	214	acetonitrile/50 mM monopotassium phosphate=20/80
CYP2C8	6-hydroxypaclitaxel	HPLC-UV	229	methanol/ammonium acetate = 27/73
CYP2C9	4-hydroxytolbutamide	HPLC-UV	230	acetonitrile/0.03% phosphoric acid = 34/66
CYP2C19	5-hydroxyomeprazole	HPLC-UV	302	acetonitrile/0.01M phosphate buffer = 25/75
CYP2D6	O-demethylation dextrorphan	HPLC-FLD	Ex=280, Em=320	phosphoric acid/ acetonitrile = 70/30
CYP2E1	6-hydroxychlorzoxazone	HPLC-UV	287	methanol/water = 55/45
CYP3A4/5	1-hydroxymidazolam	HPLC-UV	220	methanol/acetic acid = 66/34

Diamonsil C_{18} column (200 mm×4.6 mm, 5 µm) for CYP1A2, CYP2A6, CYP2B6, CYP2C8, CYP2C9, and CYP2E1 activity assays. Diamonsil C_{18} column (250 mm×4.6 mm, 5 µm) for CYP2C19, CYP2D6, and CYP3A4/5 activity assays. HPLC-UV, high performance liquid chromatography-ultraviolet. HPLC-FLD, high performance liquid chromatography-fluorescence. Ex=excitation wavelength, Em=emission wavelength.

Supplementary Table S3: The genotype distribution of *CYP2C9*3* (42614 A>C) and *CYP3A5*3* (6986A>G) in controls, HCC, fibrosis and cirrhosis groups

CYP isoforms	Allele	Groups	Total number	Genotype number (%)		
2C9	*3			**1*1*	**1*3*	**3*3*
		Control	108	103 (95.4)	5 (4.6)	0
		HCC	97	92 (94.8)	5 (5.2)	0
		fibrosis	51	49 (96.1)	2 (3.9)	0
		cirrhosis	46	43 (93.5)	3 (6.5)	0
3A5	*3			**1*1*	**1*3*	**3*3*
		Control	105	7 (6.7)	42 (40.0)	56 (53.3)
		HCC	95	8 (8.4)	42 (44.2)	45 (47.4)
		fibrosis	50	5 (10.0)	20 (40.0)	25 (50.0)
		cirrhosis	45	3 (6.7)	22 (48.9)	20 (44.4)

HCC indicates hepatocellular carcinoma.
